# Comparison between primary and secondary central nervous system vasculitis in terms of clinical, biochemical, radiological, histopathological features, and outcomes: a single-center retrospective cohort study

**DOI:** 10.3389/fneur.2025.1602427

**Published:** 2025-10-17

**Authors:** Pasquale Scoppettuolo, Lucie Pothen, Aline van Maanen, Valeria Onofrj, Selda Aydin, Olivier Gheysens, Renaud Lhommel, André Peeters, Vincent van Pesch, Halil Yildiz

**Affiliations:** ^1^Department of Neurology, Cliniques Universitaires Saint Luc, UCLouvain, Brussels, Belgium; ^2^Department of Internal Medicine and Infectious Diseases, Institute of Clinical and Experimental Research (IREC), Université Catholique de Louvain (UCLouvain), Brussels, Belgium; ^3^Statistical Support Unit, Cliniques Universitaires Saint Luc, Brussels, Belgium; ^4^Department of Radiology, Cliniques Universitaires Saint Luc, UCLouvain, Brussels, Belgium; ^5^Department of Pathology, Cliniques Universitaires Saint Luc, UCLouvain, Brussels, Belgium; ^6^Department of Nuclear Medicine, Institute of Clinical and Experimental Research (IREC), Université Catholique de Louvain (UCLouvain), Brussels, Belgium

**Keywords:** [18F]FDG-PET/CT, biopsy, stroke, PACNS, SACNS

## Abstract

**Introduction:**

Primary angiitis of the CNS (PACNS) is a rare inflammatory disorder affecting blood vessels of the brain and spinal cord causing acute stroke. This study aimed to describe clinical, biochemical, imaging and histopathological findings of a retrospective single-center PACNS cohort in comparison to a cohort of secondary angiitis of CNS (SACNS).

**Methods:**

All consecutive patients diagnosed with PACNS or SACNS between 2000 and 2023 were identified using our institutional database. Univariate comparison between both groups and multivariate analysis for independent predictors was performed, as well as Receiving Operating Characteristic analysis for white blood cell count predictive of PACNS. Kaplan–Meier curves were used for evaluating survival outcomes.

**Results:**

We identified 20 patients in each group. PACNS patients presented more frequently with seizures (40% vs. 5%, *p* = 0.02) and pseudotumoral lesions (45% vs. 10%, *p* = 0.014). In PACNS patients, median serum WBC count at diagnosis was lower (8.4×10^3^/mm^3^ [6.4–9.9] vs. 11.2×10^3^/mm^3^ [9–13.6] *p* = 0.027) and [18F]FDG-PET/CT (*p* = 0.001) was negative in all cases. No significant differences were observed for lumbar puncture profiles and diffusion weighted imaging patterns. Small-vessel vasculitis with a lymphocytic pattern was the most represented histologic phenotype in both groups. Serum WBC count ≤9.93 ×10^3^/mm^3^ was the only independent predictor of PACNS using multivariate analysis [OR 95%CI 5.107, (1.177–22.159)]. Both groups did not differ in terms of mortality, relapse and clinical outcome.

**Conclusion:**

In our study, PACNS patients presented more often with pseudotumoral lesions, seizures and small-vessel involvement and lymphocytic histologic pattern. WBC count ≤9.93×10^3^/mm^3^ was an independent predictor of PACNS diagnosis.

## 1 Introduction

Central nervous system vasculitis (CNSV) is a heterogenous group of disorders that lead to inflammation of the blood vessel wall within the central nervous system (CNS), including the brain, spinal cord and meninges, potentially resulting in ischemia and organ damage (1). CNSV may be considered as primary angiitis of the central nervous system (PACNS) in the absence of an identified underlying etiology (2) or secondary (secondary angiitis of the central nervous system, SACNS) if associated with an underlying disorder, such as systemic diseases, malignancies or infections (1).

Both entities represent a rare CNS disorder: PACNS is estimated to have an annual incidence of 2.4 cases per million person-years with Caucasian male predominance and median age of onset being 50 years (3) but it may also affect children (4). Epidemiological studies of SACNS revealed a variable proportion of CNS involvement and gender predominance according to the underlying disease (1, 5, 6). Clinical presentation is highly variable and non-specific with common clinical features including headache, encephalopathy, focal deficit or seizure (3). Therefore, diagnosis remains challenging and differential diagnosis with mimicking disorders and secondary etiologies should be ruled out through extensive laboratory work-up (7).

The 2012 Revised International Chapel Hill Consensus Conference Nomenclature of Vasculitides classified noninfectious vasculitis into three groups, large, medium and small vessel vasculitis (8). Using this nomenclature, PACNS is categorized as a single-organ vasculitis and further classified in small or medium-to-large vasculitis based on different diagnostic methods (biopsy vs. angiography) and distinct clinical characteristics and outcomes (2, 9, 10).

PACNS diagnosis relies on the diagnostic criteria established in 1988 (11). More recently, in 2009, Birnbaum et al. defined new criteria with “definite” diagnosis when CNS biopsy is available and “probable” non-biopsy diagnosis (12) only based on abnormal MRI and cerebrospinal fluid (CSF) analysis. The treatment approach is highly dependent on the subtype of CNS vasculitis. In SACNS, therapy is mainly targeted to the underlying etiology (6). In contrast, PACNS treatment generally begins with an induction therapy, consisting of high-dose intravenous corticosteroids (methylprednisolone pulse therapy 1,000 mg daily for 3–5 days) followed by oral prednisone at 1 mg/kg per day. This regimen is more commonly combined with cyclophosphamide (CYC), (13–15) but other immunomodulatory drugs can be used such as, rituximab (13, 16), infliximab (17) or tocilizumab (18). Following induction therapy, maintenance therapy is initiated with azathioprine or methotrexate (13, 14) mycophenolate mofetil (MMF) (13) and should be continued for at least 2 years before considering withdrawal in patients without recurrences (19). Treatment recommendations are primarily based on retrospective observational studies and expert opinions, as no randomized prospective data are available to guide therapeutic decisions (19). The aim of our study was to describe the clinical, biochemical, radiological, histopathological features, and outcomes of patients with PACNS in comparison to those with SACNS.

## 2 Materials and methods

### 2.1 Patient characteristics

This single center retrospective study was conducted at the tertiary hospital Cliniques Universitaires Saint-Luc in Belgium and approved by the local institutional Ethics committee (CEHF 2023/27JUL/339). All consecutive patients aged over 15 years, with a diagnosis of CNS vasculitis admitted to the Department of Internal Medicine and Neurology between January 2000 and December 2023, were retrospectively identified and reviewed. Data were collected from our institutional database (Epic Systems Corporation, Verona, WI, USA) and the database of the Internal Medicine Department. Inclusion criteria were age of >15-years-old and confirmed CNSV. The diagnosis of PACNS was based on the Calabrese and Mallek criteria (11) encompassing the presence of an unexplained acquired neurologic deficit after extensive investigation for other causes, evidence of an inflammatory arteritic process within the CNS by either angiography and/or histopathological examination without any underlying systemic disorder. In addition, we also assessed the diagnosis according to Birnbaum and Hellmann’s criteria (12) Secondary CNS vasculitis was defined as secondary involvement of the CNS as part of a systemic vasculitis due to an infectious process, a systemic inflammatory disorder, malignancy, toxin/drug exposure, radiation, or other identifiable etiologies (20).

**Figure 1:**
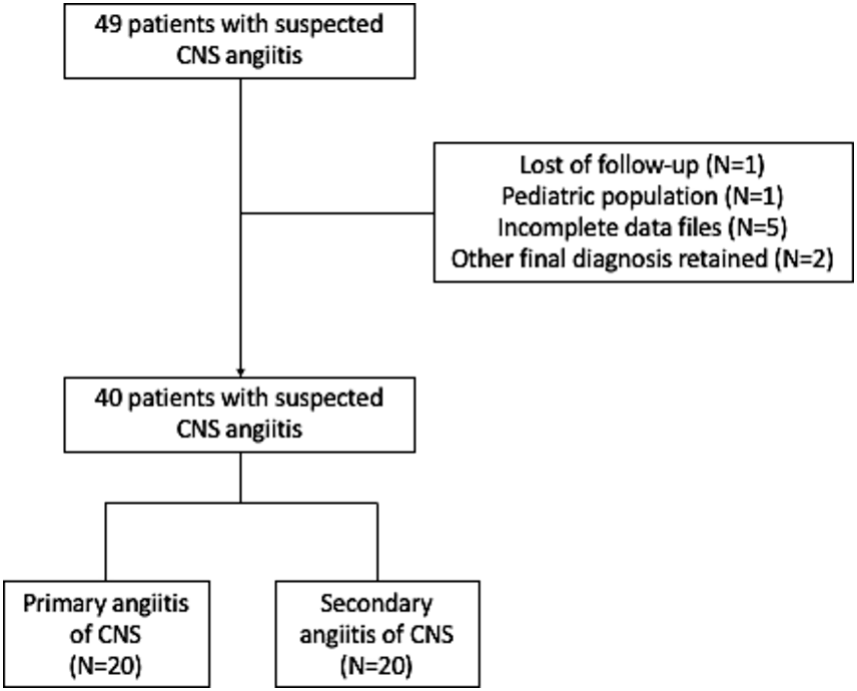
Flowchart outlining the inclusion and exclusion of patients.

Demographic and clinical characteristics of patients were collected, including age, sex, ethnic origin, past medical history, and ongoing treatment of the patients at the time of the diagnosis. Additionally, we gathered results from biological fluid tests, such as white blood cell (WBC) count, CRP level, and the presence of ANCA or ANA antibodies and serological tests (HIV, HBV, HCV, syphilis, Lyme’s disease). Furthermore, CSF analysis was conducted to determine protein (normal values [n.v.] < 45 mg/dL), glucose (n.v. 40–80 mg/dL) and lactic acid levels (n.v. 1.1–2.4 mmol/L), Gram stain, mycobacterial PCR and/or culture. In addition, the presence of CSF-specific oligoclonal bands (OCB) was investigated. We reviewed medical records for clinical symptoms at diagnosis (fever, weight loss, purpura, livedo, headache, arthralgia, cough, seizures, acute ischemic or hemorrhagic stroke, cranial nerve palsy or meningismus). Patients with acute ischemic stroke (AIS) underwent brain MRI with extracranial and intracranial imaging (CT angiography or Magnetic Resonance Angiography [MRA] to exclude other rare causes of stroke such as dissection, fibromuscular dysplasia, connective tissue disorder), cardiovascular screening (including transthoracic and transesophageal echocardiography, at least 24 h Holter ECG recording, extracranial and transcranial Doppler ultrasound). Patients with incomplete data, loss to follow-up, or uncertainty in final diagnosis were excluded from the analysis (Figure 1).

#### 2.1.1 Imaging

Brain MRI scans were acquired on a 1.5 T machine (GE Artist, GE Healthcare, Milwaukee, WI, United States) and two 3 T machines (SIEMENS Magnetom Skyra Healthineers, Erlangen, Germany; Philips Ingenia, Philips Healthcare, The Netherlands) depending on their availability at the institution.

The brain MRI protocol included axial T1-weighted imaging (slice thickness: 4 mm, gap 0.4 mm); axial T2-weighted imaging (slice thickness: 4 mm, gap 0.4 mm) T2-star-weighted imaging (slice thickness: 4 mm, gap 0.4 mm) or 3D Susceptibility Weighted Imaging (3D-SWAN (GE) slice thickness: 1.5 mm, no gap; 3D-SWI (SIEMENS and Philips) slice thickness: 1.1 mm, no gap); axial diffusion-weighted imaging (DWI) (slice thickness: 4 mm, gap 0.4 mm, B0 and B1000) with apparent diffusion coefficient (ADC) maps; axial fluid-attenuated inversion recovery (slice thickness: 4 mm, gap 0.4 mm); 3D time-of-flight (slice thickness GE: 0.6 mm; slice thickness SIEMENS and Philips: 0.5 mm); 3D T1-weighted imaging after injection of 0.2 mL/Kg of Dotarem (Gadoterate Meglumine, Gubert, France) (Slice thickness: 0.9 mm (SIEMENS) and 1 mm (GE and Philips), no gap). In a minority of patients (8/40), Vessel Wall Imaging (VWI) was acquired via a 3D Black Blood sequence after injection of Dotarem (slice thickness: 1 mm, no gap).

Imaging was reviewed for parenchymal lesions (AIS or hemorrhagic stroke) and their localization (supratentorial—cortex or deep lesions—infratentorial or both), presence of parenchymal or meningeal gadolinium enhancement, pseudotumoral lesion, and intracranial vascular abnormalities (intracranial arterial beading, stenosis or occlusion) according to previous study (21, 22) The size of involved vessels (large, medium and small) was defined according to previous criteria (23). We employed the available vascular imaging modalities—Digital subtraction angiography (DSA), CTA, or TOF/CE-MRA—in descending order of spatial resolution, to define the size of the vessels involved.

VWI was considered positive in case of typically vessel wall concentric enhancement (VWE), vessel wall thickening or perivascular enhancement previously reported and suggestive of CNSV (24).

DSA was performed through transfemoral arterial approach and selective injection of radiopaque iodinated contrast agent in carotid/vertebral arteries with a 3D representation of the rotational acquisition generated by using commercially available. 3D angiography software (Syngo InSpace 3D) was transferred to a dedicated computer workstation for postprocessing (Siemens).

#### 2.1.2 Nuclear imaging

18F-fluorodeoxyglucose-positron emission tomography computed tomography ([18F]FDG-PET/CT) acquisition was performed on a Philips Gemini TF 16/64 or a Philips Vereos (Philips Healthcare, Cleveland, OH, United States). All patients fasted for at least 4 h and fasting blood glucose levels were less than 150–200 mg/dL prior to intravenous administration 3–4 MBq/kg of FDG. After FDG administration, patients were instructed to rest in a quiet room. Whole-body imaging with or without dedicated brain acquisition was performed 60 min after FDG administration. A low-dose CT-scan was performed for attenuation correction and whole-body PET emission data were acquired from the vertex to mid-thighs or feet.

#### 2.1.3 Pathological examinations

For diagnostic work-up, skin, lung, kidney or temporal artery biopsy was performed when indicated, at the discretion of the clinician. Brain biopsies were either MRI-guided, targeting a radiologically abnormal area, or performed as a blinded procedure, typically from the non-dominant frontal or temporal lobe. The resulting biopsy fragment was fixed in formalin and embedded in a paraffin block. Hemalun-eosin staining was performed, and in some cases serial sections were necessary. Diagnostic histopathological features of PACNS included transmural inflammation affecting leptomeningeal or parenchymal vessels. As previously reported, three types of vascular inflammatory involvement were considered: lymphocytic (inflammatory infiltrate consisting of lymphocytes), granulomatous (inflammatory infiltrate with granulomas) and acute necrotizing pattern (vascular wall undergoing fibrinoid necrosis) (25).

#### 2.1.4 Treatment and clinical outcomes

The treatment in the acute phase (steroids, CYC, and tocilizumab) and long-term immunosuppressive therapy (methotrexate, azathioprine, MMF, cyclosporine, and anti-TNF monoclonal antibodies) was retrieved from the electronic records. Opportunistic infections, cancer incidence and antibiotic treatment were documented as well. Clinical outcomes were reported as: relapse, defined as the recurrence of neurological symptoms or new AIS on MRI; good clinical outcome, defined by a score 0–2 on the modified Rankin Scale (mRS) (26), in comparison with poor outcome, indicated by a mRS of 3–6 at 1 year. Mortality was also recorded.

### 2.2 Statistical analysis

Non-parametric reporting was used for continuous parameters (median values and 25th–75th percentiles) as data were not normally distributed (Shapiro–Wilk test, QQ plot). Categorical data were reported as incidence and percentages. Formal statistical tests were used to compare demographic and radiological findings and clinical outcome between the PACNS and SACNS groups using Fisher’s exact test or Mann–Whitney U-test according to the parameter type and distribution. The receiver and Operating Curve (ROC) method was used to determine the optimal WBC cut-off for PACNS. Univariate and multivariate logistic regression modeling was used to identify prognostic factors for PACNS. The higher bound for the inclusion of candidate variables in the multivariate model was set to 10%, and backward stepwise selection was used to select the optimal model. Overall survival (OS) was defined as the time from the date of diagnosis to the date of death due to any cause. For patients who did not die (i.e., those who were lost to FU or who were alive at the date of data cutoff), the time to death was censored at the time of the last FU. Kaplan–Meier estimate of OS were presented with 95% confidence intervals (CIs), minimum and maximum. OS rates were also be calculated at various timepoints. All *p*-values were 2-tailed, and *p* values of less than 0.05 were considered statistically significant. The analysis was performed using SAS software (version 9.4; SAS Institute Inc., Cary, NC, United Sates).

## 3 Results

### 3.1 Study population

Forty patients with CNS angiitis were identified. Twenty patients were diagnosed with PACNS (50%), and 20 with secondary CNS vasculitis (50%) (Figure 1). Size of vessels involvement according to the primary or secondary involvement of CNS is reported in Supplementary Table 1.

### 3.2 PACNS cohort

Median age was 54 years [44.5–63], and 12 patients were male (60%). Detailed clinical symptoms at diagnosis, radiological and biological (serum and CSF) findings, and treatment regimen as well as clinical outcome and complication are presented in Table 1. Noteworthy 50% of patients presented with AIS on MRI, and lumbar punction was abnormal in 17/19 patients (89%).

**Table 1:** Comparison between primary (PACNS) and secondary angiitis of the central nervous system (SACNS) patients for demographics, clinical, radiological characteristics.

Demographics	PACNS (*N* = 20)	SACNS (*N* = 20)	*P*-value
Age at diagnosis (years)	54 [44.5–63]	64 [38–74]	0.310
Sex (male)	12 (60%)	11 (55%)	0.999
Ethnicity			0.498
Caucasian	16 (80%)	15 (75%)	
Asian	1 (5.3%)	3 (15%)	
Sub-saharian	1 (5.3%)	2 (10%)	
African	2 (10.5%)	0	
Symptoms			
Stroke	10 (50%)	16 (80%)	0.096
Headache	10 (50%)	7 (35%)	0.523
Seizure	8 (40%)	1 (5%)	0.020
Weight loss	5 (25%)	8 (40%)	0.501
Meningismus	5 (25%)	4 (20%)	0.999
Fever	3 (15%)	8 (40%)	0.155
Arthralgia	3 (15%)	4 (20%)	0.999
Cranial nerve palsy	2 (10%)	1 (5%)	0.999
Livedo	1 (5%)	2 (10%)	0.999
Purpura	0	2 (10%)	0.487
Cough	0	1 (5%)	0.999
Any symptom	18 (90%)	18 (90%)	0.999
CRP level (mg/L) at diagnosis	5.7 [1–8.8]	13.8 [2–40.7]	0.153
Blood WBC level (×10^3^/mm^3^) at diagnosis	8.4 [6.4–9.9]	11.2 [9–13.6]	0.027
Lumbar puncture	19 (95%)	14 (70%)	
CSF cells/mm^3^	7 [2.5–17]	25.5 [4–155]	0.266
CSF proteins (mg/dl)	53 [35–80.5]	69 [40–94]	0.999
OCB analysis in CSF	15 (75%)	7 (35%)	
Presence of OCB in CSF	3 (15%)	1 (5%)	0.605
Abnormal CSF analysis	17/19 (89%)	10/14 (58%)	0.363
Cerebral MRI characteristics			
Acute ischemic stroke lesions	10 (50%)	15 (75%)	0.191
Supratentorial	8 (40%)	12 (60%)	0.343
Infratentorial	2 (5%)	5 (25%)	0.407
Both	1 (5%)	3 (15%)	0.605
Atrophy	7 (35%)	3 (15%)	0.273
Periventricular /White Matter Hyperintensities	13 (15%)	11 (55%)	0.748
Microbleeds	3 (15%)	4 (20%)	0.999
MRA beading	3 (15%)	5 (25%)	0.678
Proximal stenosis or occlusion	2 (10%)	5 (25%)	0.407
Distal stenosis or occlusion	1 (0.5%)	3 (15%)	0.605
Anterior	2 (10%)	5 (25%)	0.408
Posterior	1 (0.5%)	2 (10%)	0.999
Leptomeningeal enhancement	8 (40%)	4 (20%)	0.301
Parenchymal enhancement	10 (50%)	6 (30%)	0.333
Suggestive findings of vasculitis on MRI	8 (40%)	9 (22.5%)	0.999
Hemorrhagic stroke			
Parenchymal hemorrhage	1 (5%)	2 (10%)	0.999
SAH	1 (5%)	1 (5%)	0.999
Pseudotumoral lesion	9 (45%)	2 (10%)	0.014
VWI (N = 9)			
Performed	5 (62.5%)	4 (50%)	
Abnormal findings	4 (50%)	2 (25%)	0.661
DSA performed	8 (4%)	1 (5%)	
Abnormal DSA	2 (1%)	0	
[^18^F]FDG-PET/CT Whole Body	18 (90%)	14 (70%)	0.235
Abnormal [^18^F]FDG-PET/CT Whole Body	0 (0%)	10 (50%)	<0.001
Biopsies	16 (80%)	10 (50%)	
Brain	15 (75%)	6 (30%)	
Temporal artery	0	2 (10%)	
Skin	1 (5%)	1 (5%)	
Lung	0	1 (5%)	
Phenotype			
Biopsy-diagnosed	12 (60%)	6(30%)	
Imaging-diagnosed	8 (40%)	14 (70%)	
Diagnosis according to Birnbaum and Hellmann Criteria			
Definite	13 (65%)	N. A	
Probable	7 (35%)	N. A	
Treatment			
Corticosteroids	19 (95%)	17 (85%)	0.605
Cyclophosphamide	7 (35%)	4 (20%)	0.480
Azathioprine	9 (45%)	6 (30%)	0.514
Methotrexate	2 (10%)	4 (20%)	0.661
Mycophenolate mofetil	3 (15%)	3 (15%)	0.999
Anti-infectious agent (antibiotics, antiviral)	1 (5%)	3 (15%)	0.605
Rituximab	3 (15%)	1 (5%)	0.605
Tocilizumab	0	2 (10%)	0.487
Chemotherapy	0	2 (10%)	0.487
Colchicine	0	1 (5%)	0.999
Anti-TNF	0	1 (5%)	0.999
Outcome			
Neurologic sequelae	13 (65%)	9 (45%)	0.341
Follow-up	50 [17–76]	14 [5.5–56]	0.114
1-year mRS	3 [2–6]	2 [1–6]	0.879
mRS 0–2	8 (40%)	11 (55%)	0.999
mRS 3–5	2 (10%)	3 (15%)	0.999
Death	7 (35%)	6 (30%)	0.999
Relapse	2 (10%)	5 (25%)	0.407
Opportunistic infection	3 (15%)	5 (25%)	0.695
Cancer	0	0	NA

Continuous variables are reported as median with 25th–75th percentiles and categorical parameters as incidence and percentage.

[18F]FDG-PET/CT Whole-Body, 18F-fluorodeoxyglucose-positron emission tomography computed tomography Whole-Body; ANA, antinuclear antibody; ANCA, antineutrophil cytoplasmic antibodies; DSA, digital substraction angiography; MPO, Myeloperoxidase antibodies; PR3, Anti-proteinase-3; mRS, Modified Rankin Scale; MRI, magnetic resonance imaging angiography; MRI, magnetic resonance imaging; OCB, oligoclonal band; SAH, Subarachnoid hemorrhage; TNF, Tumor Necrosis Factor; VWI, Vessel wall-Imaging; NA, Not Available.

### 3.3 SACNS cohort

SACNS cohort included 5 patients with giant cell arteritis (GCA) (25%), 3 with ANCA-associated vasculitis (15%), 3 with infection-related vasculitis, 2 cases of Pneumococcal meningitis-associated vasculitis and one case of VZV-associated vasculitis (15%), 3 with hemopathy (2 cases of Chronic Lymphocytic Leukemia and one case of T-cell lymphoma) (15%), 3 with connective tissue disorder-associated (CTD) (one case each of Sjögren’s syndrome, systemic lupus erythematosus, and Alport’s syndrome) (15%), 2 with Behçet’s disease (10%) and 1 with Takayasu arteritis (5%).

### 3.4 Comparison between PACNS and SACNS

. Demographic and ethnic characteristics did not differ between the two groups. In contrast, PACNS patients presented significantly more often with epileptic seizures (40% vs. 5%, *p* = 0.02), and a lower median blood WBC level (5.7 ×10^3^/mm^3^ [1–8.8] vs. 13.8 ×10^3^/mm^3^ [2–40.7], *p* = 0.027) compared to SACNS patients. CSF analysis results did not differ between the two groups in terms of WBC count, protein levels or oligoclonal band detection.

Univariate logistic analysis to assess the association between clinical findings and PACNS diagnosis showed that seizures (odds ratio [OR] of 12.667 [1.402–114.410], *p* = 0.024), as well as low serum WBC count (OR 0.771 [0.61–0.974], *p* = 0.029) at the diagnosis were associated with PACNS diagnosis (Table 2).

**Table 2:** Univariate logistic analysis for PACNS.

Predictor	Number of patients	Likelihood ratio (DF)	Odds ratio (95% CI)	*p*-value
CRP	37	2.106 (1)	0.987 (0.969–1.005)	0.147
Seizure	40	5.112 (1)	12.667 (1.402–114.410)	0.024
Fever	40	2.942 (1)	0.265 (0.058–1.209)	0.086
Leptomeningeal enhancement	39	1.333 (1)	2.333 (0.554–9.83)	0.248
Acute ischemic stroke	40	3.750 (1)	0.250 (0.061–1.017)	0.053
Acute ischemic stroke on MRI	39	2.065 (1)	0.37 (0.096–1.436)	0.151
Vasculitis findings on MRI	39	0.003 (1)	0.889 (0.250–3.156)	0.856
WBC count	35	4.752 (1)	0.771 (0.61–0.974)	0.029

DF, Degree of freedom; OR, odds ratio; WBC, White blood cells; 95% CI, 95% Confidence interval.

ROC curve identified a WBC count cut-off of 9.93 × 10^3^/mm^3^ (Area Under the Curve of 0.719 [*p* = 0.01], accuracy for the corresponding Youden index of 0.36 with 75% sensitivity and 61% specificity) for the prediction of PACNS (Table 3). In multivariate analysis, a serum WBC count lower than the threshold was an independent predictor of PACNS with an OR of 5.107 (*p* = 0.029) [1.177–22.159].

**Table 3:** Multivariable analysis: independent predictors for primary vasculitis.

	Wald chi-square (DG)	*P*-value	OR (95%CI)
WBC ≤9.93 ×10^3^/mm^3^	4.72	0.0029	5.107 (1.177–22.159)

CI, confidence interval; DG, Degree of freedom; OR, Odds-ratio; WBC, White blood cells.

**Figure 2:**
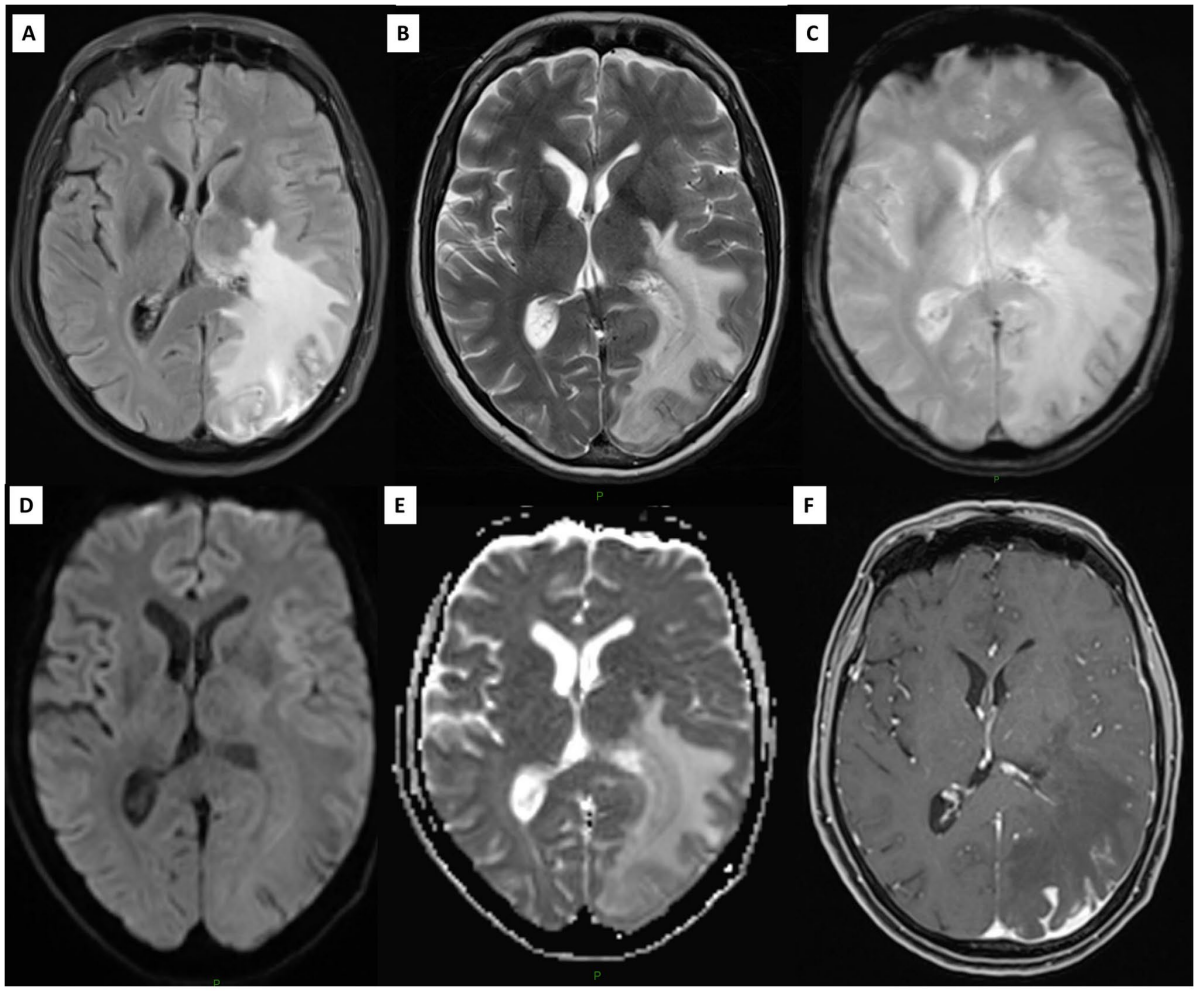
Brain MRI showing a pseudotumoral lesion in the left hemisphere. **(A)** Axial FLAIR and **(B)** T2-weighted imaging. **(C)** T2*-weighted imaging demonstrates a small intralesional hemorrhage **(D,E)** DWI and ADC confirm vasogenic edema of the lesion. **(F)** After gadolinium injection, parieto-occipital cortico-pial contrast enhancement is observed on the left side, with corresponding pachymeningeal thickening.

**Figure 3:**
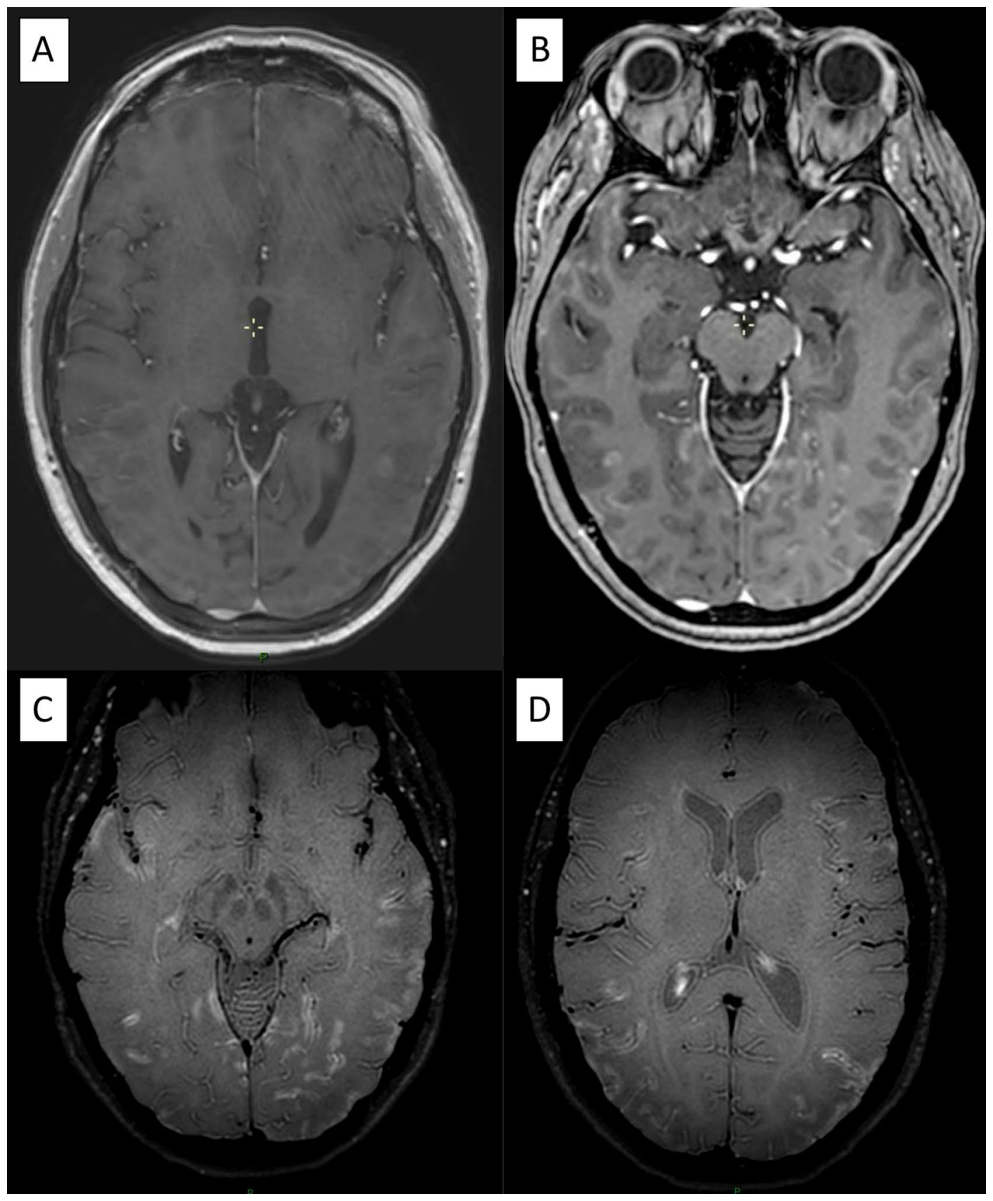
A 40-year-old patient with biopsy-proven small vessel vasculitis. **(A)** Post-gadolinium 3D T1-weighted MRI shows parenchymal enhancement in the parietal lobe and associated leptomeningeal enhancement **(B)**. **(C and D)** VWI demonstrates diffuse and extensive leptomeningeal contrast enhancement.

Imaging analysis revealed more often the presence of a pseudotumoral lesion at diagnosis in PACNS (45% vs. 10%, *p* = 0.014) and all patients with PACNS had negative [18F]FDG-PET/CT (*p* < 0.001) (Figure 2). No differences were found in the presence of atrophy, periventricular/white matter hyperintensities, microbleeds, intracranial arterial beading or stenosis location on MRA. Small-size vessel involvement was the most representative pattern in both primary and secondary CNS angiitis (Figure 3 and Supplementary Table 1). For PACNS, 12 cases were biopsy-diagnosed and 8 imaging-diagnosed. For SACNS, 6 cases were biopsy-diagnosed and 14 imaging-diagnosed.

**Figure 4:**
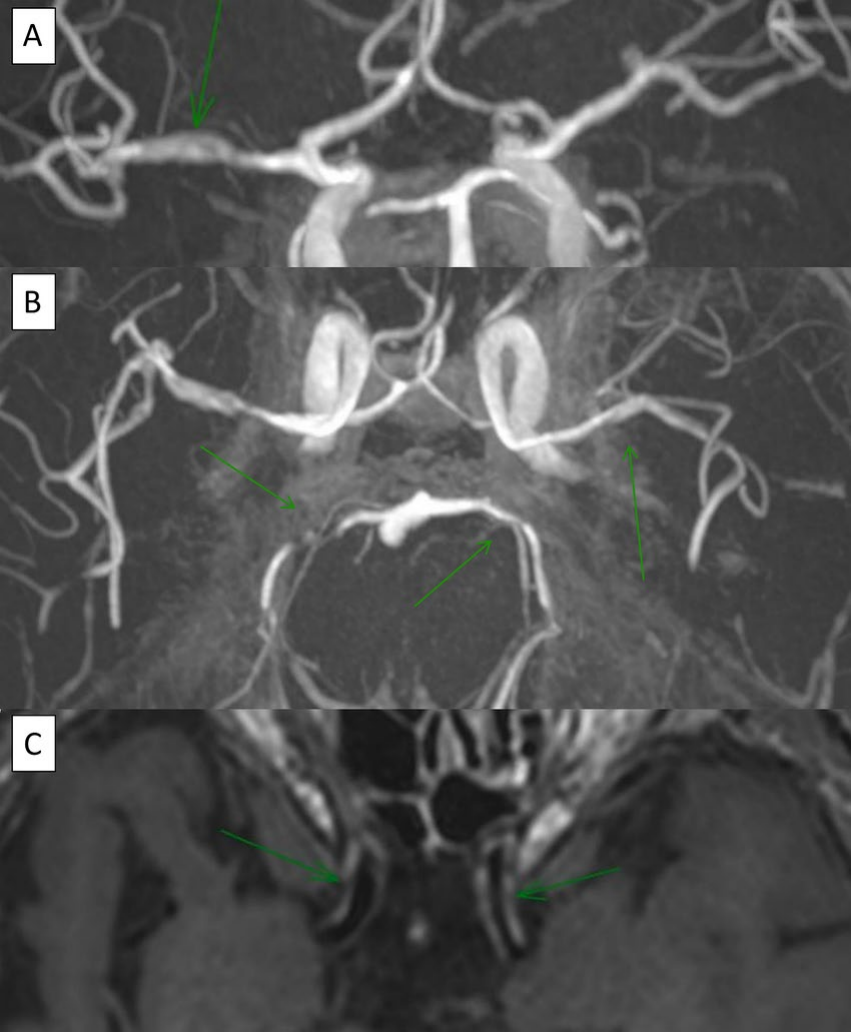
A 70-year-old patient with medical history of multiples acute ischemic stroke. Time-of-flight (TOF) brain MRI reveals multiple intracranial stenoses (**A**, coronal view; **B**, axial view), while vessel wall MRI (VWI) demonstrates circumferential contrast enhancement (**C**, axial view).

VWI showed wall enhancement of the main and smaller trunks of the circle of Willis (Figure 4), with segmental vascular irregularities visualized on TOF in 4 PACNS patients and 2 SACNS (*p* = 0.37) Further details and biopsy correlation (when available) are provided in the Supplementary Table 2. For PACNS, VWI positive findings were reported in one case of small, one case of mediumand two cases of a combination of small and medium-vessel vasculitis. Interestingly, in one patient with PACNS there was a negative biopsy with positive VWI imaging and another patient had negative VWI imaging but positive biopsy (small-vessel involvement and lymphocytic pattern) (Supplementary Table 2). For SACNS, 2 patients presented with positive findings (large-vessel vasculitis) and 2 patients presented with negative findings (one small-vessel and one large-vessel vasculitis), but no brain biopsies were available.

**Figure 5:**
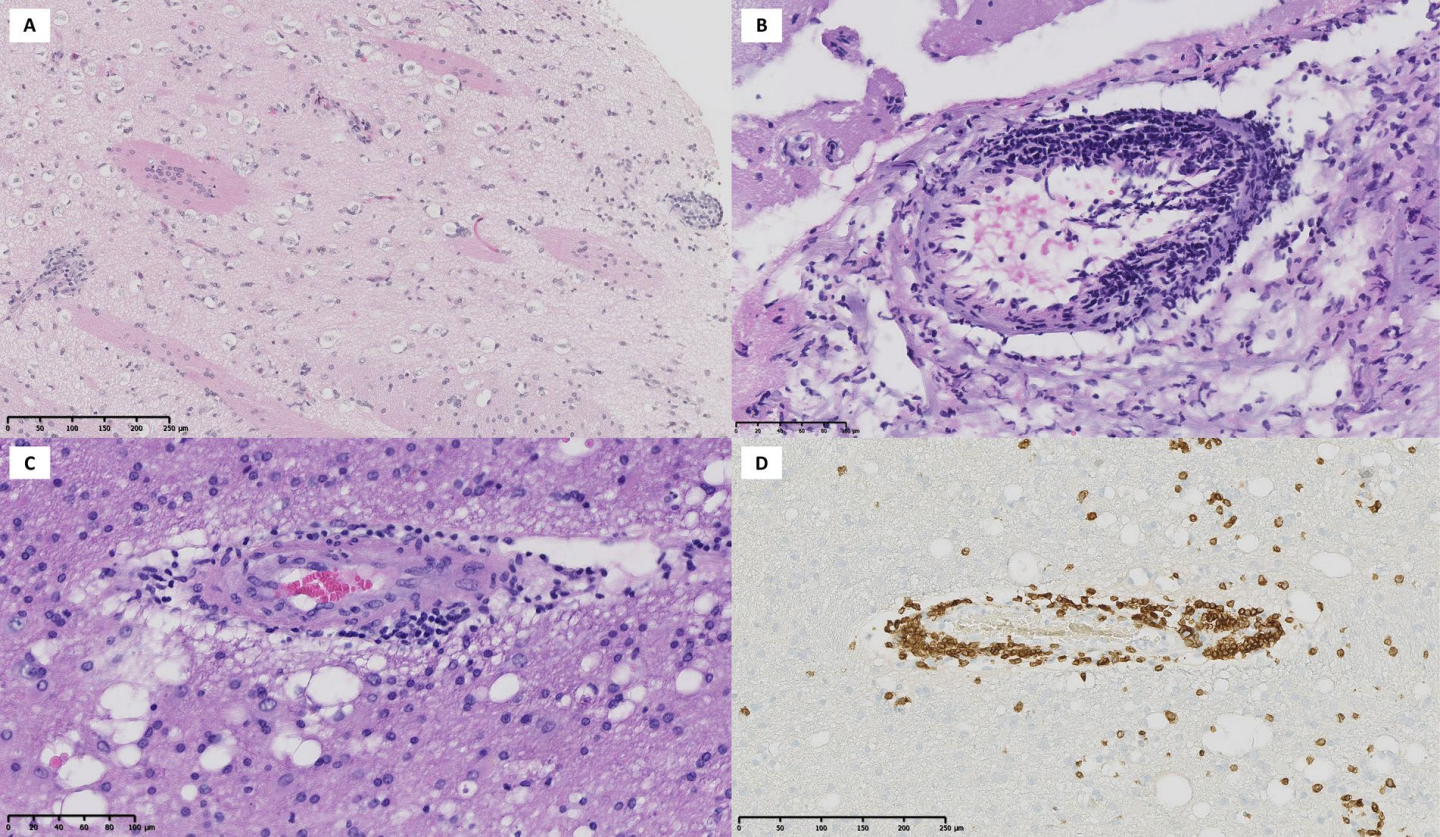
**(A)** Brain parenchyma and leptomeningeal arteries, hematoxylin–eosin, 11×. **(B)** Eccentric and transmural infiltration of arterial vessel, hematoxylin–eosin, 27×. **(C)** Arterial wall infiltration with lymphocytes (red blood cells in the lumen), hematoxylin–eosin, 21×. **(D)** CD3 staining of the same artery confirming lymphocytic infiltrate, 15×.

Overall tissue biopsy was obtained in 26 patients (65%). For most cases, the biopsy site was the brain (21/40, 52.5%), followed by the skin and temporal artery (2/40, 5%), and in only one case, the lung (2.5%). Arterial biopsies of the skin and temporal regions were negative. Lung biopsy was compatible with small-vessel involvement, consistent with ANCA-associated vasculitis in one patient with SACNS. Histology of brain biopsies showed a prevalent lymphocytic phenotype in both groups (Figure 5) but results of 4 biopsies (19%) were negative or not contributive (Supplementary Table 3). No patient experienced serious complications due to biopsy. Eight biopsies were performed blindly, 11 were MRI-guided, and the method was not specified in two cases.

Seven cases of opportunistic infection were observed during FU: one case of Aspergillus, one of COVID-19 and Pseudomonas, one ENT infection without an identified pathogen, one CMV infection, one combined infection due to Stenotrophomonas and *Enterococcus faecalis* and two cases of *Streptococcus pneumoniae* infection. We did not observe any cancer occurrence during the FU period, except for the three cases of hematologic malignancies associated with SACNS.

**Figure 6:**
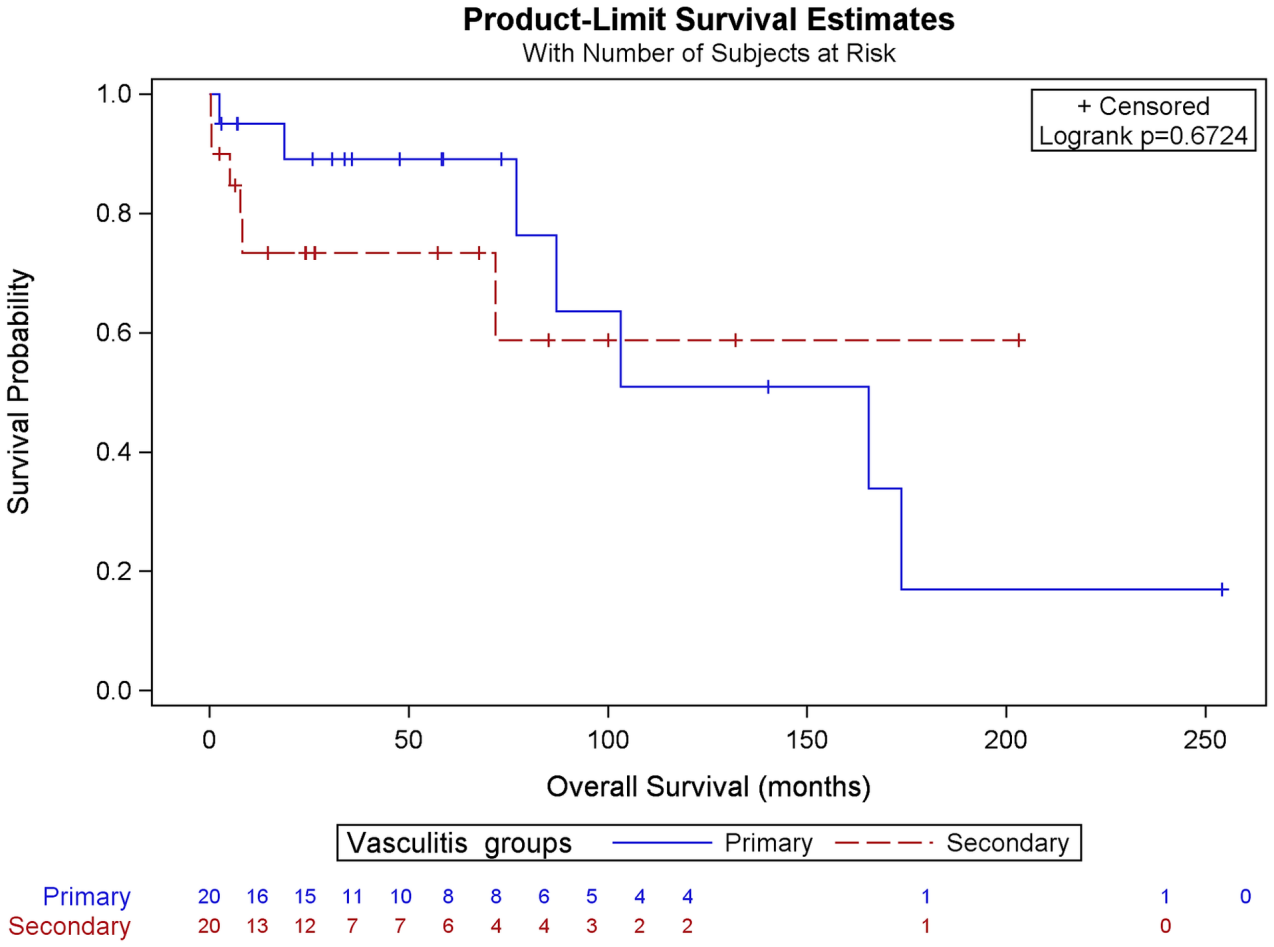
Kaplan–Meier estimator for primary and secondary vasculitis.

There was no difference in the treatment administered and clinical outcomes at FU between the two groups. Kaplan–Meier analysis showed a median FU of 50 [17–76] for PACNS and 14 [5.5–56] for SACNS, with no significant differences between two groups (*p* = 0.114) in terms of overall survival (Figure 6), and including all causes of mortality.

## 4 Discussion

Comparison of PACNS with SACNS revealed that seizures and pseudotumoral lesions were prevalent clinical characteristics in PACNS. Additionally, a low serum WBC count (≤9.93 ×10^3^/mm^3^) was identified as an independent predictor of PACNS with an OR of 5.10, a sensitivity of 75% and specificity of 61%. No differences in lumbar puncture results were noted between the two groups. Radiologically, PACNS presented more frequently with pseudotumoral lesions but rates of intracranial arterial abnormalities, contrast enhancement and VWI abnormalities were similar compared to SACNS. Small-vessel involvement and a lymphocytic histological pattern on biopsy were the most reported findings in both groups. There were no significant differences in clinical outcomes, including rate of relapse, median mRS at 3-months FU, or mortality between both groups.

PACNS and SACNS are both a rare disorder with neither specific nor pathognomonic symptoms (27). Clinical manifestation may be similar and challenging for clinicians (2). Initial assessment encompasses serum and serological studies in order to exclude a secondary etology (4). Acute phase reactants (ESR and CRP) are generally recognized as negative in PACNS (5, 28). Our finding of a normal WBC count (reference range 4–10 ×10^3^/mm^3^ in our laboratory) in CNS angiitis should prompt clinicians to consider PACNS. However, a comprehensive laboratory work-up is still recommended (6). Additionally, a study comparing ICAD and PACNS found no statistically significant differences in terms of ESR or CRP, indicating that these serum markers are not useful for diagnosis other forms of intracranial arteriopathy (29).

Vascular imaging findings in PACNS did not significantly differ from those observed in SACNS. While the proportion of acute ischemic stroke (AIS) was higher in the SACNS group, the prevalence of parenchymal or vascular abnormalities was similar between the two groups. Notably, leptomeningeal and parenchymal enhancement were more frequently observed in the PACNS group, likely reflecting the high prevalence of small vessel vasculitis and pseudotumoral presentations in our cohort. VWI was performed in a comparable number of patients across both groups, revealing typical findings such as concentric vessel wall enhancement, wall thickening, or perivascular enhancement (24). A recent study (30), despite its small sample size, reported a trend toward proximal arterial involvement and anterior circulation predominance in PACNS, whereas SACNS showed a tendency for distal and both anterior/posterior involvement. However, given our similarly limited sample size and the relatively low frequency of small vessel vasculitis with intracranial involvement, we did not observe any consistent pattern in the localization of abnormalities.

[18F]FDG-PET/CT may indirectly contribute to the diagnosis of PACNS, by excluding underlying etiologies as shown in our cohort. [18F]FDG-PET/CT is a well-established imaging tool for evaluating malignancies and its use in inflammatory and infectious disorders has grown rapidly in the past decade as has the body of evidence-based literature. Nowadays, [18F]FDG-PET/CT is considered the method of choice for most infectious and inflammatory disorders and has been adopted in several clinical recommendations and guidelines (31). Indeed the EULAR task force recently updated its recommendations regarding the role of [18F]FDG-PET/CT in diagnosing large-vessel vasculitis (32). In addition to the well-known value of the [18F]FDG-PET/CT to assess the extracranial arteries, the EULAR 2023 recommendations state that [18F]FDG-PET/CT can be used as an alternative to ultrasound for assessing cranial arteries in patients with suspected GCA. These recommendations are supported by the sensitivity and specificity for cranial arteries reported by multiple studies: a systematic literature review and meta-analysis found a good diagnostic performance in GCA, with a pooled sensitivity of 58% (95%CI 45–71%) and specificity of 97% (95%CI 91–99%) (33). Furthermore, [18F]FDG-PET/CT is a well-recognized diagnostic tool in for identification of SACNS etiologies, such as underlying hematological disorders or infections (34).

The clinical outcome was good in 40% of the patients with PACNS and 55% of patients with SACNS, with a median mRS of 3 and 2, respectively., We noted a mortality rate of 35% for PACNS and 30% for SACNS. Compared to other cohorts of PACNS, our mortality rate was higher probably due to an older population and the longer FU, despite the small proportion of medium/large-vessel involvement and the prevalent lymphocytic pattern, which have been associated with a better outcome (19, 28). The mRS at last FU was a median of 3, likely due to the high prevalence of small-vessels PACNS and in line with small-vessel cohorts outcome already reported (10, 35). These results confirmed two clinical features associated with a high disability score at last FU—age and stroke- as reported in a previous cohort (14). Concerning SACNS, available data are heterogeneous and largely influenced by the underlying etiology. A systematic review reported a higher mortality rate among patients with ANCA-associated vasculitis compared to other forms of systemic vasculitis (36). In cases of infectious vasculitis, mortality rates vary considerably depending on the causative pathogen (37).

We reported 20 new cases of PACNS, adding to the existing literature, and providing further insights into its clinical spectrum, imaging characteristics, and diagnostic challenges, particularly in relation to small-vessel involvement and the role of advanced neuroimaging techniques. Clinically PACNS patients presented with AIS, headache and seizures as the most prevalent symptoms. This finding is consistent with previous findings from other PACNS cohort studies (9, 28, 35, 38, 39). However, our cohort exhibited less MRA stenosis or beading on MRI, likely because of a higher proportion of patients with small-vessel involvement. Compared with other series, where AIS and medium-sized vessel involvement were more prevalent (21, 22, 28), we observed a higher frequency of clinical features such as seizures at onset, as well as radiological findings including pseudotumoral lesions and leptomeningeal enhancement, patterns expected in a cohort dominated by small-vessel PACNS (10, 35). Notably, we report a high rate of biopsy-proven cases, which exceeds that of larger cohorts with similar small-vessel predominance (60%). Most of these cases demonstrated a lymphocytic infiltration pattern on histology (9, 28, 39). This aligns with the distinct phenotype described in biopsy-confirmed small-vessel PACNS, which is associated with specific clinical and paraclinical features (9, 10, 28, 35). These include less frequent ischemic strokes and vascular beading, and more frequent occurrences of seizures, abnormal CSF findings, pseudotumoral lesions, and leptomeningeal enhancement, features also evident in our cohort.

The vast majority of patients had abnormal CSF parameters. Despite the CSF findings in our cohort mirroring those of previous studies (10, 28, 35) and being considered non-specific and non-contributory, recent ESO Guidelines (19) recommended CSF analysis for differential diagnosis and exclusion of infection and neoplasm. These guidelines confirm that a normal test does not exclude the diagnosis, given its low sensitivity and limited accuracy.

Half of PACNS patients presented with acute ischemic stroke (AIS) and lesions on MRI. We found a very low incidence of intracranial bleeding compared to the reported in the literature (9, 21), with only one case of subarachnoid hemorrhage and one case of intracerebral hematoma. This could be explained by the absence of the necrotizing histological subtype and its lower prevalence in female patients (40), both of which are associated with hemorrhagic presentation. In addition, we reported a high proportion of radiological pseudotumoral lesions in our cohort, which may indicate a selection bias toward cases more likely to be biopsy-diagnosed. At our center, we noted no complications and only four cases of non-contributive biopsy, despite a reported risk of serious complication being up to 4% (41). Small-vessel involvement may explain the low rate of digital subtraction angiography performed at our center for imaging-proven cases of PACNS. Due to its variable sensitivity and specificity for vasculitis (4, 42) and poor accuracy in detecting small-vessel involvement (4, 10), DSA is increasingly replaced by intracranial vessel imaging (43). Given the good concordance found with MRA-TOF (44), only eight DSA were performed, of which two were positive and both were attributed to medium-vessel vasculitis.

The main parameters of interest in VWI are concentric wall thickening and vessel-wall enhancement (VWE) (24). Concentric wall thickening, although highly prevalent in patients with PACNS, can also be observed in reversible constriction syndrome (RCVS) and other pathologies (45). VWE is not specific for PACNS, as it has been detected in normal vessel segments of PACNS patients (46), other pathologies (24) and even in healthy controls (47). Additionally, the rate of arterial stenosis co-localizing with VWE varies (22, 45, 48). Despite promising results, the use of VWI remains limited and debated. The lack of standardized protocols, hardware differences, and variable timing with respect to diagnosis or treatment have hindered its broader adoption. Further studies are needed to clarify its accuracy across vessel sizes and histological patterns in PACNS. In our cohort, both PACNS and SACNS, we found negative results in case of small-vessel angiitis and positive findings in case of medium/large-vessel angiitis. In particular, within our cohort of PACNS, one patient had a positive VWI and negative biopsy (likely due to medium-size vessel involvement, that was not sampled) while another patient had a negative case of VWI and a positive biopsy (Figure 3), presumably reflecting very small-vessel involvement below the resolution of MRI. This confirms an inverse relationship between the size of the vessel involved and VWE, consistent with previous findings (22, 49), which demonstrate less frequent abnormalities in small-vessel angiitis or biopsy-diagnosed cases.

Finally, in our cohort, all the patients had at least one abnormal test result among MRI, CSF analysis and biopsy. This confirms the previous finding that while normal findings alone do not exclude the diagnosis of PACNS, the combination of negative results from several ancillary tests may exclude the diagnosis (12, 50). Almost all our patients received corticosteroids, and nearly half were treated with intravenous CYC during the initial phase, aligning more with the French cohort’s protocol (13, 39). The low relapse rate observed may be attributed more to high mortality than to treatment efficacy. Although some evidence suggests that corticosteroids alone may achieve prolonged remission without relapses (19), at our center we tend to initiate immunosuppressive therapy (particularly CYC) in severe cases to reduce steroid-related toxicity. Given the potential severity of PACNS, this approach aligns with ESO recommendations, which support the use of immunosuppressive agents in more severe presentations. Azathioprine was the most frequently used maintenance therapy. However, the small sample size limited comparisons across vessel phenotypes or treatment regimens in relation to complications.

To the best of our knowledge, this is the first study comparing a cohort of PACNS and SACNS patients. In clinical practice, pseudotumoral lesions with seizures, an absence of inflammatory syndrome, and a negative [18F]FDG-PET/CT result should suggest PACNS to clinicians and prompt the appropriate work-up and treatment.

The strengths of our study include a multidisciplinary approach to diagnosis, a large number of biopsy-proven cases, and long-term FU, which allowed for confirmation of the diagnosis and exclusion of mimics such as CNS lymphoma and RCVS.

However, this study has some limitations. This is a retrospective and cross-sectional study with a small sample size for both groups, and the single-center design raises concerns regarding about potential referral bias. Furthermore, despite excluding PACNS mimics through an extensive work-up, ancillary examinations and imaging modalities (MRI, DSA and VWI) are not part of the standard protocol of investigations. Instead, these tests rely on the physician’s clinical suspicion which may evolved over time. The absence of standardized protocols limits the ability to compare modalities or analyze accuracy compared to the clinical diagnosis of CNS vasculitis as reference. Finally, the absence of data on the time interval from onset to diagnosis is a further limitation, as it may have contributed to poor outcomes in some cases.

## 5 Conclusion

This retrospective single-center study reported 20 cases of PACNS and compared them to 20 cases of SACNS from the same academic hospital. Patients with PACNS more frequently presented with seizures and pseudotumoral lesions, and had a lower serum WBC count, which was identified as an independent predictor of PANCS. All patients with PACNS patients had a negative [18F]FDG-PET/CT scan. Clinical outcomes were similar between groups. Small-vessel vasculitis and lymphocytic inflammation were the most common patterns. Future research with larger cohorts, standardized protocols for advanced imaging techniques, including comparison with biopsy when available, and longer FU periods will be essential to enhance understanding and optimize the differential management of PACNS and SACNS.

## Data Availability

The raw data supporting the conclusions of this article will be made available by the authors, without undue reservation.
