# Supplementary material for: Comparison between primary and secondary central nervous system vasculitis in terms of clinical, biochemical, radiological, histopathological features, and outcomes: a single-center retrospective cohort study
Source: Front Neurol. 2025 Oct 17;16:1602427. doi: 10.3389/fneur.2025.1602427 (PMC12575130; doi:10.3389/fneur.2025.1602427)
Supplement: Supplementary file 1 [file Supplementary_file_1.pdf]

Supplementary Material

1 Supplementary Tables

Supplementary Table 1

Size of vessels involvement according to the phenotype of the vasculitis (see text and reference [24]).

Size of vessels involvement	PACNS	SACNS
Small	14/20	11/20
Medium	3/20	3/20
Large	0/20	6/20
Combination of sizes	3/20	0

Supplementary Table 2

VW-MRI in PACNS and SACNS cohort.

PACNS: Primary Angiitis of Central Nervous System; SACNS: Secondary Angiitis of Central Nervous System; VW-MRI: Vessel Wall MRI.

**VW-MRI**		
PACNS	**Positive findings (N=4)**	**Negative findings (N=1)**
Negative brain biopsy	1/2 (50%); Combination of small and medium size vessels	0
Positive brain biopsy	1/2 (50%); Small vessels size, necrotic pattern	1/1; Small vessels size, lymphocytic pattern
Small vessel vasculitis	1/4	1/1
Medium vessel vasculitis	1/4	0
Large vessel vasculitis	0/4	0
Combination of patterns	2/4	0
SACNS	**Positive findings (N=2)**	**Negative findings (N=2)**
Negative brain biopsy	NA	NA
Positive brain biopsy	NA	NA
Small vessel vasculitis	0	1/2
Medium vessel vasculitis	0	0
Large vessel vasculitis	2/2	1/2
Combination of patterns	0	0

Supplementary Table 3

Histology of brain biopsy

PACNS: Primary Angiitis of Central Nervous System; SACNS: Secondary Angiitis of Central Nervous System

Histology of brain biopsy	PACNS (N=15)	SACNS (N=6)
Granulomatous	2/15	0
Lymphocytic	7/15	2/6
Necrotic	1/5	1/6
Combination of patterns	2/15	2/6
Negative	3/15	1/6
